# Using whole-genome sequences of the LG/J and SM/J inbred mouse strains to prioritize quantitative trait genes and nucleotides

**DOI:** 10.1186/s12864-015-1592-3

**Published:** 2015-05-28

**Authors:** Igor Nikolskiy, Donald F Conrad, Sung Chun, Justin C Fay, James M Cheverud, Heather A Lawson

**Affiliations:** Department of Genetics, Washington University School of Medicine, Campus Box 8108, 660 S Euclid Ave, St Louis, MO 63110 USA; Division of Genetics, Brigham and Women’s Hospital and Harvard Medical School, Boston, MA USA; Department of Biology, Loyola University, Chicago, IL USA

**Keywords:** Mouse models, Quantitative trait loci (QTL), Complex traits, Identity-by-descent (IBD), Predicted deleterious mutations, Quantitative trait gene (QTG), Quantitative trait nucleotide (QTN), Candidate loci, Whole genome sequence, Small nucleotide variant (SNV)

## Abstract

**Background:**

The laboratory mouse is the most commonly used model for studying variation in complex traits relevant to human disease. Here we present the whole-genome sequences of two inbred strains, LG/J and SM/J, which are frequently used to study variation in complex traits as diverse as aging, bone-growth, adiposity, maternal behavior, and methamphetamine sensitivity.

**Results:**

We identified small nucleotide variants (SNVs) and structural variants (SVs) in the LG/J and SM/J strains relative to the reference genome and discovered novel variants in these two strains by comparing their sequences to other mouse genomes. We find that 39% of the LG/J and SM/J genomes are identical-by-descent (IBD). We characterized amino-acid changing mutations using three algorithms: LRT, PolyPhen-2 and SIFT. We also identified polymorphisms between LG/J and SM/J that fall in regulatory regions and highly informative transcription factor binding sites (TFBS). We intersected these functional predictions with quantitative trait loci (QTL) mapped in advanced intercrosses of these two strains. We find that QTL are both over-represented in non-IBD regions and highly enriched for variants predicted to have a functional impact. Variants in QTL associated with metabolic (231 QTL identified in an F_16_ generation) and developmental (41 QTL identified in an F_34_ generation) traits were interrogated and we highlight candidate quantitative trait genes (QTG) and nucleotides (QTN) in a QTL on chr13 associated with variation in basal glucose levels and in a QTL on chr6 associated with variation in tibia length.

**Conclusions:**

We show how integrating genomic sequence with QTL reduces the QTL search space and helps researchers prioritize candidate genes and nucleotides for experimental follow-up. Additionally, given the LG/J and SM/J phylogenetic context among inbred strains, these data contribute important information to the genomic landscape of the laboratory mouse.

**Electronic supplementary material:**

The online version of this article (doi:10.1186/s12864-015-1592-3) contains supplementary material, which is available to authorized users.

## Background

The LG/J (large) and SM/J (small) strains of inbred mice were independently derived from selection experiments for large and small body size at 60 days, respectively [1]. LG/J was created from a pool of albino mice obtained from a commercial breeder over a nine-month period and selected for increased body size for over 50 generations [2,3].

SM/J was created from a pool of mice derived from four crosses of seven inbred strains: dilute brown agouti (*dba*), silver chocolate (*sv ba*), black and tan (*a*^*t*^), pink-eyed, short-eared dilute brown agouti (*ps*^*e*^*dba*), albino (*c*), cinnamon spotted (*bs*), and agouti (*a*) [4]. It is unclear whether any of these strains are related to current laboratory inbred strains. The SM/J strain is kept heterozygous at the *agouti* locus by mating black animals (*a/a*) with their white-bellied agouti (*A*^*w*^*/a*) siblings. Attempts to fix the SM/J strain or its Recombinant Inbred Strain offspring for the SM/J allele at *agouti* have resulted in strain failure [5].

Subsequent to selection, the LG/J and SM/J strains are fully inbred and have been maintained at the Jackson Laboratory since the 1950s by brother-sister mating. They are at the extremes of the adult body size distribution among the common laboratory inbred strains and have been profitably studied for genetic variation in adult body size and growth [6-18]. Early genetic studies of these two strains found the differences in body size are caused by many genes of individually small effects [8]. This result has been confirmed in many quantitative trait locus (QTL) mapping studies. The strains have remained phenotypically stable over time, except that the SM/J strain is now about 6g heavier than it was in Chai’s studies [19].

LG/J and SM/J differ in many complex traits in addition to size and growth. The parental strains and their crosses differ in skeletal morphology, including the size and shape of the skull [20-22], mandible [23-30], tooth morphology [31,32], long-bone lengths [33-37], and a variety of other skeletal elements as well as bone biomechanical and structural properties [38,39].

They also differ for a variety of metabolic traits including obesity, diabetes, and serum cholesterol, triglycerides, and free fatty acids levels [40-51]. The SM/J strain responds more strongly than LG/J to a high-fat diet for these metabolic traits. The two strains also differ for maternal genetic effects on offspring growth and offspring adult metabolic traits [52-56] and their cross has been useful in mapping parent-of-origin genetic effects on metabolic traits [48-51,53,57].

In addition to these diverse metabolic and skeletal phenotypes, the LG/J strain shows the rare ability to regenerate tissues after injury. The LG/J strain regenerates ear pinna tissues after a 2mm hole-punch [58-61] while the SM/J strain does not. The LG/J strain also can regenerate damaged articular cartilage [62] and is protected from post-traumatic osteoarthritis [63].

Others have mapped a variety of behavioral phenotypes using a LG x SM cross, including prepulse inhibition [64] and methamphetamine sensitivity and locomotor activity [65]. Additional studies have investigated blood cell parameters [66] and skeletal muscle weight and fiber types [67,68].

Here we describe the whole-genome sequences of the LG/J and SM/J inbred mouse strains, adding two more sequences to the collection of whole-genomes of laboratory mice [69,70]. We integrate these sequences with quantitative trait loci (QTL), and illustrate how top-down (phenotype to genomic sequence) and bottom-up (genomic sequence to phenotype) approaches can be used to identify candidate quantitative trait genes (QTG) and prioritize positional candidate quantitative trait nucleotides (QTN) for further mechanistic studies. Results from QTL studies using crosses of LG/J and SM/J will be more interpretable given these whole-genome sequences.

## Results and discussion

### LG/J and SM/J whole-genome sequences

**Figure 1:**
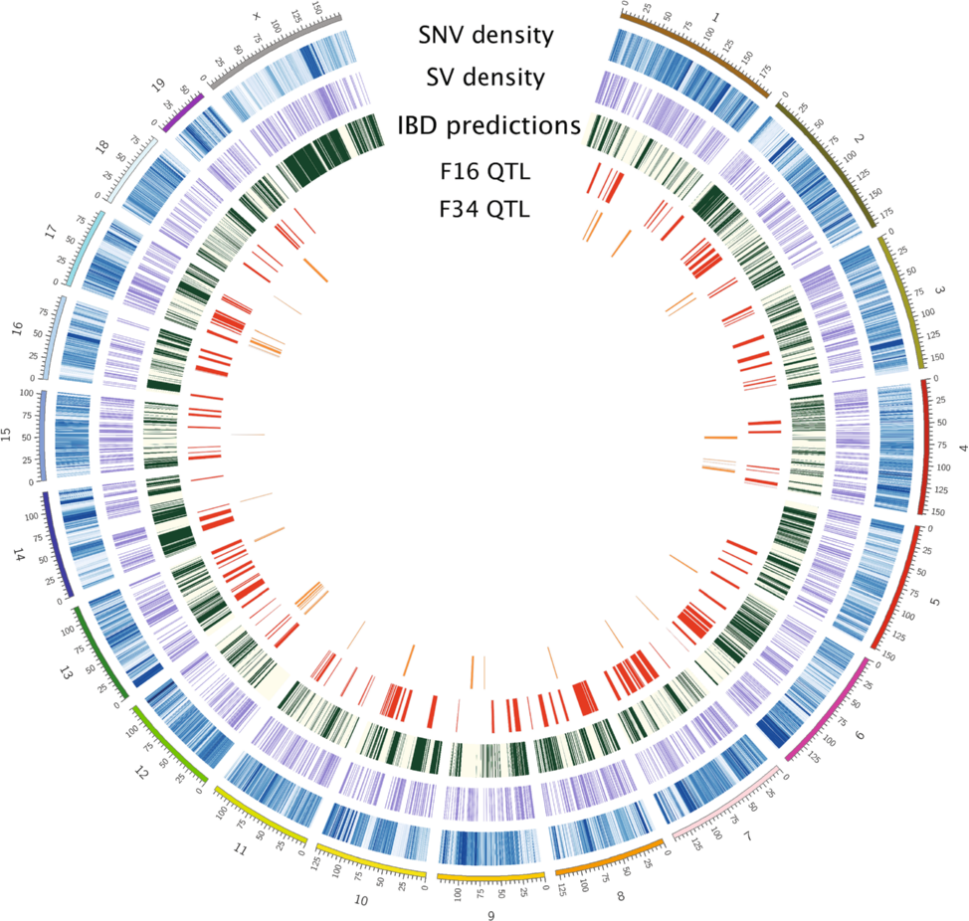
Circos plot illustrating the integration of polymorphic SNVs, SVs, and regions that are predicted to be identical-by-descent (i.e. harboring little to no variation) between the LG/J and SM/J inbred strains. These data are combined with QTL for metabolic traits mapped in an F_16_ advanced intercross between LG/J and SM/J and with QTL for bone-growth traits mapped in an F_34_ advanced intercross between these two strains.

Sequence was generated from DNA isolated from 1 LG/J female and 1 SM/J female as described below. Variants (comprised of small nucleotide variants (SNVs) including single nucleotide polymorphisms (SNPs) and small insertion-deletions (Indels) and structural variants (SVs) including deletions, insertions and inversions) were discovered based on whether they were the same as, or different from, the C57BL/6J reference sequence (mm10, NCBI build 38). Greater than 90% of reads for each strain could be uniquely mapped to the reference genome and, based on a genome size of 2.5G, our coverage is approximately 35X and 30X for LG/J and SM/J, respectively. Overviews of annotated variants identified for each strain individually, as well as those that are polymorphic between LG/J and SM/J, are described in Tables 1 and 2 and in Additional files 1 and 2. SNVs identified for the LG/J strain are available in Additional files 3 and 4 and for the SM/J strain in Additional files 5 and 6. Structural variant positions and classifications for each strain are available in Additional file 7. Polymorphic variants and their genomic context are illustrated in Figure 1. Quality was assessed by comparing our SNP calls with SNPs in dbSNP that were called from unpublished low-coverage sequence generated independently via two separate library preparations for LG/J (≈18X) and SM/J (≈14X) (Table 3).

**Table 1:** **Sequence and variants overview**

**Strain**	**Total reads**	**% Mapped to reference**	**Coverage**	**SNPs**	Strain-Specific SNPs*	**Indels**	Strain-Specific Indels*	**SVs**	Strain-Specific SVs*
LG/J	9.6x10^8^	91.72	35X	4,663,723	146,814	1,127,072	56,469	12,802	1,361
SM/J	7.7x10^8^	92.67	30X	4,792,568	227,132	1,127,458	65,935	15,564	2,255

^*^Refers to strain-specific variants with respect to 18 other available mouse genome sequences.

**Table 2:** **Genomic overview of structural variants**

**Variant**	**LG/J**	**SM/J**	Polymorphic*
Deletion	7,697	9,889	11,206
Insertion	5,038	5,590	7,698
Inversion	94	159	184

^*^Refers to polymorphisms between LG/J and SM/J.

**Table 3:** **Comparison of high-coverage SNPs to low-coverage sequence in dbSNP**

	**LG/J**	**SM/J**
Number of matched positions and variants	3,693,085	3,798,743
Number of mismatched variants at matched positions	20,266	44,582
Number of SNPs in dbSNP not called in high coverage genomes	411,859	391,672
New SNP variants identified in high coverage sequence	942,372	941,383

We compared SNPs identified in LG/J and SM/J to those identified in other mouse strains sequenced at high coverage and identified novel variants (Table 1). Pairwise distances were calculated for LG/J and SM/J from each of these strains and a phylogenetic tree was constructed using all 20 mouse sequences (Additional files 8 and 9). The LG/J and SM/J ancestries are expected given what we know about their origins and previous studies of inbred mouse phylogenies [71]. It has been suggested that the SM/J strain may be related to the current DBA strains, DBA/1J and DBA/2J, as one of the seven strains contributing to the base population that was selected for smaller body weight was a *dba* strain named after its dilute brown agouti coat color [72]. Comparison of SNPs between LG/J or SM/J and the sequenced DBA/2J shows no specific relationship between SM/J and DBA/2J. However, the percent shared SNPs between inbred mouse strains can be biased by the use of C57BL/6J as the reference genome since variants may be missed due to alignment quality favoring bases that match the reference.

### Functional predictions

**Figure 2:**
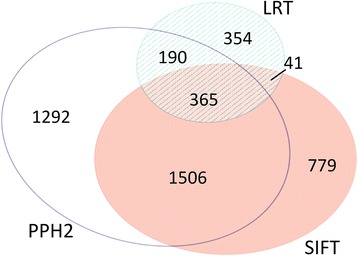
Proportional Venn diagram illustrating the intersections of three independent methods of predicting a functionally damaging amino acid changing SNP between the LG/J and SM/J strains.

Deleterious amino acid mutations in LG/J and SM/J were predicted using three independent methods: LRT [73], PolyPhen-2 [74] and SIFT [75] as implemented in VEP [76]. The LRT compares the probability that a codon has evolved under a conserved model to the probability that a codon has evolved under a neutral model. The conserved model allows a codon to have evolved under negative selection and the neutral model assumes the rates of nonsynonymous and synonymous mutations are not significantly different. PolyPhen-2 uses both sequence- and structure-based predictive features to characterize the functional importance of a mutation, making use of non-redundant protein databases. SIFT is based on the principles of protein evolution and has been applied to a variety of organisms ranging from bacteria to humans. The algorithm is based on sequence homology and uses a median conservation score to measure protein conservation. The numbers of deleterious amino acid predictions for each strain are listed in Table 4 and predicted scores are provided in Additional file 10. The intersections of these predictions for SNPs that are polymorphic between LG/J and SM/J are illustrated in Figure 2. It is worth noting that the three different methods give largely non-overlapping results, which is consistent with a similar analysis using human genomes [73]. This result implies that our ability to reliably annotate whole genome sequences is still somewhat lacking.

**Table 4:** **Variants of predicted functional importance**

	LRT	PolyPhen2	SIFT	Highly Informative	Highly Informative
				TFBS (SNPs)	TFBS (Indels)
LG/J	800	2,748	2,198	578	61
SM/J	928	2,907	2,271	629	60
Polymorphic*	950	3,353	2,691	662	90

^*^Refers to polymorphisms between LG/J and SM/J.

In addition to SIFT predictions, VEP identifies variants falling in noncoding and potentially regulatory regions that may impact a gene’s expression. An overview of potentially functional regulatory variants is listed in Table 4 and positions and scores are provided in Additional files 11 and 12.

### LG/J, SM/J identical by descent regions

We identified genomic regions that are highly conserved between LG/J and SM/J using a hidden Markov model (Figure 1 and Additional file 13). These regions are defined as stretches of sequenced DNA ≥ 50kbp in length containing little to no polymorphism between the strains. These regions are likely to be identical by descent (IBD), and are non-randomly distributed throughout the genome, being more clustered than expected by chance (p < 2.2e^−16^, Wald-Wolfowitz test). We classified 39% of the LG/J and SM/J sequenced genomes as IBD.

### LG x SM QTL and whole-genome sequences

We integrated all of these data – IBD regions, polymorphisms and functional predictions – with 272 published QTL for metabolic traits (obesity, diabetes and serum-lipids) and for bone-growth traits (femur, humerus, radius and tibia length) mapped in very advanced intercrosses of LGxSM [37,48-51]. LOD scores range from 2.4 to 11.89 and are significant for their respective study. Additional file 14 describes these QTL, including their genomic coordinates, and their integration with various sequence characterizations. Figure 1 illustrates these QTL in relation to the sequence parameters we have generated. We find that 27% of all QTL interrogated in this study are covered by IBD bases, whereas 39% would be expected if they were randomly distributed. We find that both trait-specific QTL peaks (the point of the QTL with the lowest probability of a chance association with a specific trait) and QTL-specific peaks (accounting for pleiotropy, where more than one trait maps to a locus) are over-represented in non-IBD regions (*χ*^2^ = 42.6, df = 1, p = 6.6x10^−11^ for trait-specific peaks and *χ*^2^ = 16.8, df = 1, p = 4.03x10^−05^ for QTL-specific peaks). Further, we find that QTL regions are more likely to harbor variants that are predicted to have a functional impact by comparing the number of amino-acid changing variants predicted to be damaging by at least one algorithm and the number of variants predicted to fall in high-impact positions of transcription factor binding sites to empirical distributions of numbers generated from 1000 sets of randomly chosen non-QTL, non-IBD regions of similar size (p < 2.2e^−16^ for both amino acid changing and TFBS variants, Additional file 15).

**Figure 3:**
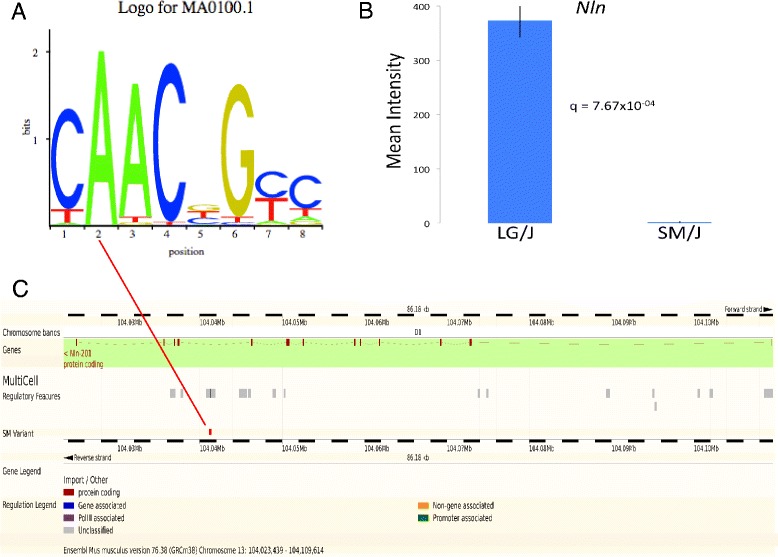
Connecting a putative quantitative trait nucleotide (QTN) with a candidate quantitative trait gene (QTG). **A**: A SNP identified in the SM/J strain (chr13:104041257 A→C) falls in a highly informative position (position 2) of a predicted cMyb TFBS. **B** and **C**: This SNP falls in an intron of *Nln*, a protein-coding gene associated with gluconeogenesis. *Nln* falls in a QTL associated with variation in basal glucose levels in an F16 generation of a LG x SM advanced intercross and is highly significantly differentially expressed between the LG/J and SM/J strains. **C**: This variant overlaps multiple regulatory elements and is a strong candidate for mechanistic studies of *Nln* function in glucose metabolism.

Integrating these data provides an opportunity to examine variation between LG/J and SM/J from both bottom-up (genomic sequence to phenotype) and top-down (phenotype to genomic sequence) perspectives with the goal of identifying plausible quantitative trait nucleotides (QTN) for testing mechanistic hypotheses. To illustrate a bottom-up approach, we focus on a SNP identified in the SM/J strain (chr13:104,041,257 A→C) falling in a cMyb TFBS (JASPAR ID MA01001) with a predicted high-impact (Figure 3). This position overlaps regulatory elements including H3K36 and H3K4 histone marks and a DNase1 hypersensitive site (ENSMUSR00000276453). The SNP falls in an intronic region of the oligopeptidase neurolysin, *Nln* (NM_029447). *Nln* knockout mice have been shown to be more insulin sensitive and glucose tolerant and to have increased gluconeogenesis relative to littermate controls [77]. In mammals, the liver is the main site of gluconeogenesis. A microarray analysis of hepatic gene expression shows *Nln* to be highly significantly differentially expressed between LG/J and SM/J, with the SM/J strain’s expression barely detectable [78]. This gene falls within the support intervals of a QTL associated with basal serum glucose levels in an F_16_ generation of an advanced intercross (AI) between the LG/J and SM/J strains [50]. Thus *Nln* is an attractive candidate quantitative trait gene (QTG) and this SNP is an attractive quantitative trait nucleotide (QTN) for further mechanistic studies of *Nln* involvement in glucose metabolism.

**Figure 4:**
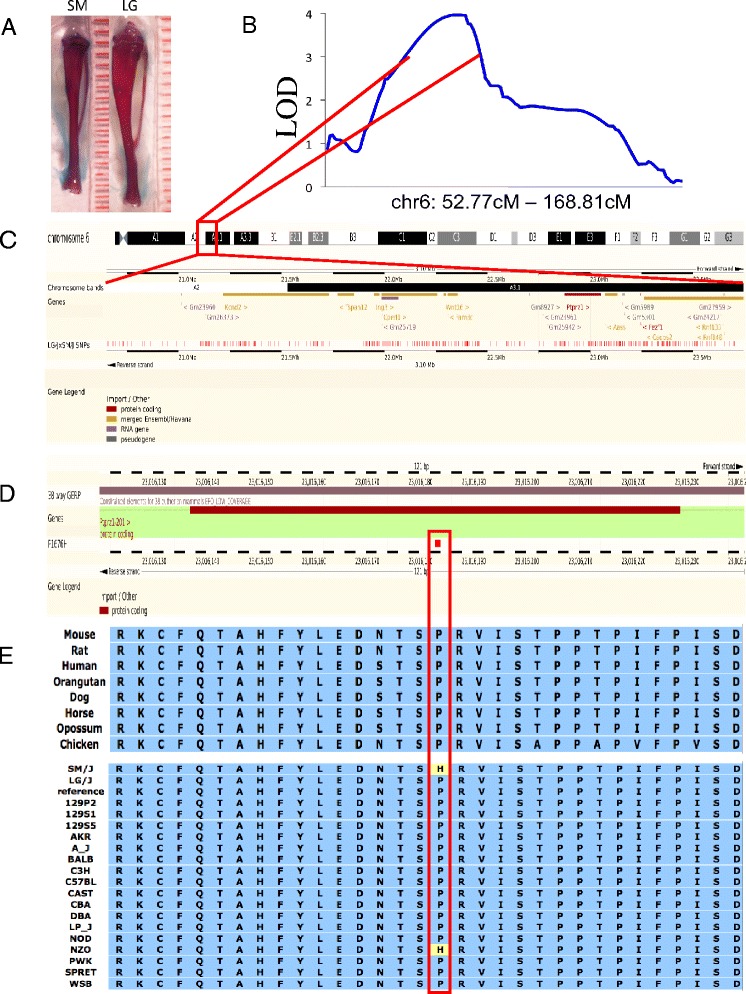
Connecting phenotypic variation (QTL) to a candidate quantitative trait gene (QTG) and putative quantitative trait nucleotide (QTN). **A** and **B**: Variation in tibia length (mm) between LG and SM mapped to a QTL on chromosome 6. **C**: This QTL was localized to a genomic region and intersected with LG/J, SM/J SNPs and IBD regions. **D**: SNPs falling in non-IBD regions were interrogated and a SNP falling in exon 14 of *Ptprz1*, which affects bone-growth, was identified. The SNP changes the encoded amino acid from proline to histidine. **E**: This amino acid is 100% IBD and the amino acid variant occurs in SM/J and one other unrelated strain of laboratory mouse, NZO/HlLtJ. This SNP is predicted to be functionally damaging by the LRT, PolyPhen-2 and SIFT algorithms, and represents a fruitful candidate QTN for further functional follow-up.

To illustrate a top-down approach, we focus on a QTL mapped in an F_34_ generation of the LGxSM AI that is associated with variation in tibia length at chr6: 20,650,821-23,746,386 (Figure 4). There are 12 protein coding genes and 10 RNA genes falling within the QTL support interval [37]. Two of these genes, *Wnt16* (NM_053116) and *Ptprz1* (NM_001081306), are involved in limb development and/or bone formation [79,80]. Variants in a third gene, *Cped1* (NM_001081351), have recently been associated with variation in bone mineral density in a GWAS [81]. 23% of this QTL is covered by bases falling in IBD regions, but these three genes are located in non-IBD portions of the QTL. Three nonsynonymous SNPs fall in *Cped1* and are predicted to have functional consequences by at least one of the algorithms used: LRT, PolyPhen or SIFT. A fourth nonsynonymous SNP is predicted to have functional consequences by all three algorithms making it a highly attractive QTN. The SNP falls in exon 14 of *Ptprz1* (ENSMUSE00000619494, position 23016230 C→A; P1676H). This exon is highly conserved. Sanger sequencing confirmed the SM/J variant and the amino acid changing polymorphism between the strains. *Ptprz1* encodes the protein R-PTP-Z, which is thought to modulate osteoblast metabolism through dephosphorylating *Src*, which plays a key role in osteoblast activities such as adhesion and differentiation [82,83]. The amino acid change occurs in the SM/J strain, and the only other strain of sequenced mouse carrying this variant is NZO/HlLtJ. NZO/HlLtJ is a common laboratory strain used as a model of metabolic syndrome because of its extreme obesity and hyperglycemic phenotype [84]. It bears no special relationship with SM/J, despite sharing this particular variant and a similar metabolic phenotype on a high-fat diet.

## Conclusions

Here we describe the whole-genome sequences for the LG/J and SM/J inbred mouse strains. LG/J and SM/J are frequently compared in QTL mapping studies because of their great phenotypic diversity, and because this diversity is normally distributed in intercrosses of these two strains. This makes LG x SM an ideal model system for studying the genetic architecture of normal variation in complex traits, because this most closely mimics that found in human populations, where most variation is the result of many interacting genes of individually small effects. Comparison with previous imputation methods using SNPs called from low-coverage sequence for LG/J and SM/J indicated that there were many unreported variants in these strains [51,85]. Our sequence provides higher resolution, which allows us to capture more of the genomic sequence variation found in these strains with greater certainty.

Because we are interested in identifying candidate variants for functional follow-up, our first order of business was to identify regions of the LG/J and SM/J genomes that do not vary between the strains. Identification of IBD genomic regions is a powerful way to narrow existing QTL support intervals, as the regions that do not vary between the strains are unlikely to cause variation in the mapped trait. Further, identification of IBD and non-IBD regions between two strains is a powerful tool for testing mechanistic hypotheses and for planning focused candidate gene studies [86]. We classified 39% of the LG/J and SM/J sequenced genomes as IBD, and we are able to use this classification to narrow QTL support intervals by orders of magnitude when eliminating stretches of DNA that contain little to no variation between the parental strains within QTL support intervals. Eliminating these regions does not explicitly rule them out as harboring potentially causal variants, however focusing on the non-IBD sequence within QTL support intervals – especially on the variants predicted to have functional consequences – allows one to prioritize the so-called ‘low-hanging fruit’. Other commonly used strain pairs, such as C57BL/6J and DBA/2J, have been sequenced but have not been subject to such close evaluation of IBD genomic regions within QTL.

We integrated the rich source of QTL results generated by very advanced intercrosses of LG x SM for metabolic traits (231 QTL associated with variation in obesity, diabetes and serum-lipids) mapped in the F_16_ and for bone-growth traits (41 QTL associated with variation in femur, humerus, radius and tibia) mapped in the F_34_ generations with the LG/J and SM/J genomic sequences. The median QTL interval in the F_16_ data is ≈ 3.5mB and for the F_34_ it is ≈ 2.0mB (Additional file 14). We find that ≈ 29% of F_16_ and ≈ 19% of F_34_ QTL intervals are covered by IBD regions. Subtracting these IBD regions from QTL reduces the genomic search space for both generations, resulting in a comparable ≈ 1.2mB median number of per QTL bases for both F_16_ and F_34_ generations. We find that QTL from both generations have proportionally equivalent numbers of SNVs, bases covered by SVs, and bases predicted to have functional consequences (Additional file 14). Further, we find that QTL regions as a class are more likely to harbor variants predicted to have functional consequences relative to randomly selected non-QTL and non-IBD genomic regions of similar size (Additional file 15).

The traits associated with the QTL interrogated here belong to different classes of quantitative phenotype, namely metabolic traits mapped in the F_16_ generation and developmental traits mapped in the F_34_ generation. Having QTL for the same trait replicate in multiple generations of a mapping study increases the probability that the QTL is causal. However, given the high costs (both financial and time-wise) of breeding and maintaining large populations of mice for many generations, incorporating whole-genome sequence parameters as we have illustrated here can be used to extract compelling information from QTL mapped in earlier generations of intercrosses, even when the support intervals span many mega-bases of sequence. Thus whole-genome sequence data should be made part of the toolkit used to inform the design, execution and follow-up of QTL mapping studies.

We have highlighted variants falling in a QTL on chromosome 13 associated with basal glucose levels and a QTL on chromosome 6 associated with tibia length (Figures 3 and 4) to illustrate how all of these data – whole genome sequence variants, IBD regions, QTL, and functional predictions – can be integrated with each other and with other public datasets to identify and prioritize QTG and QTN. Identifying such variants within QTL in mice can facilitate translational research for correlated traits in human studies, and potentially uncover genetic underpinnings of disease phenotypes [87,88]. Our initial description and analysis of the LG/J and SM/J whole genomes offers new data that can be used to address fundamental questions about the molecular nature of quantitative phenotypic variation in an important model system.

## Methods

### Ethics statement

All animal care and handling procedures conformed to IACUC guidelines.

### DNA isolation and library construction

DNA was isolated from the livers of one adult female LG/J and one adult female SM/J mouse using the Qiagen DNAeasy Blood and Tissue kit (Qiagen, West Sussex, UK). Genomic DNA was sonicated to an average size of 175 bp. The fragments were blunt ended, had addition of “A” base to 3’ end, and had Illumina’s sequencing adapters ligated to the ends. The ligated fragments underwent amplification incorporating a unique indexing sequence tag. The resulting libraries were sequenced on 6 lanes using the Illumina HiSeq-2500 as paired end reads extending 101 bases from both ends of the fragments.

### Alignment and SNV detection

Reads were aligned to the GRC m38 (mm10) mouse reference genome using NovoAlign-2.08.02 and a BAM file was produced for each strain (http://www.novocraft.com).

The gene annotation model used was Ensembl *Mus musculus* GRC 38.72. Variants were called from each strain’s BAM file using two variant discovery tools: SAMtools pileup and FreeBayes Bayesian genetic variant detector [89,90]. Results were merged and only variants with a minimum read depth of 3 and a quality score of at least 20 for SNPs and at least 50 for indels were included in the final set of SNVs for analysis.

### Quality assessment and Sanger sequencing candidate variant

SNPs identified for LG/J and SM/J were compared to SNPs called from independently generated, low-coverage sequence available on dbSNP [91] using custom python scripts. DNA was isolated from the livers of 4 female LG/J and 4 female SM/J animals to validate the candidate amino acid changing mutation P1676H in exon 14 of *Ptprz1*. The following primers were designed: Forward 5’-GCT CCA TGG CCA CTA TCT TTA CTC-3’ and reverse 5’-CAA TTC ATG CCT CAA GGT GAC TGC-3’. Sanger sequencing was performed by Genewiz (South Plainfield, NJ).

### Comparison with variants identified in other sequenced mouse strains

VCF files for SNPs discovered in 18 mouse strains’ whole-genomes were downloaded (http://www.sanger.ac.uk/resources/mouse/genomes) and compared to LG/J and SM/J SNPs using custom python scripts.

### Structural variant prediction

Structural variants (SV) were identified using a local implementation of the SVmerge pipeline [92]. SVmerge simplifies SV discovery and integration using multiple SV discovery algorithms, producing a single consensus SV callset. The SVmerge pipeline was run with 4 calling algorithms: Breakdancer 1.0, pindel 0.2.4q, SECluster and cnD [92-95]. After merging the results of these 4 callers into a single consensus callset, the Velvet genome assembler was used to attempt breakpoint assembly for all SVs in the callset [96].

### Deleterious mutation prediction

#### LRT

Deleterious amino-acid changing mutations were predicted using a likelihood ratio test (LRT) as described in Chun and Fay (2009). The algorithm was modified to compare the genomes of LG/J and SM/J each to the reference C57BL/6 J. P-values were calculated by comparing twice the log-likelihood ratio of the two models to a χ^2^ distribution with df = 1. Deleterious amino acid changing mutations were predicted using an LRT cutoff of p < 0.001 while controlling for false positives and false negatives as previously described and modified for mouse genomic sequence [73].

#### PolyPhen-2

PolyPhen-2 was downloaded and run locally to predict deleterious mutations in the LG/J and SM/J genomes. The algorithm supports analysis of mouse proteins using prebuilt human models if the option ‘-n mouse’ is specified when preparing the local copy of UniProtKB (www.uniprot.org). Deleterious amino acid changing mutations were predicted for both strains using a recommended false discovery rate (FDR) cutoff of 20% [74].

#### VEP and SIFT

VEP was downloaded and run locally to predict deleterious mutations in the LG/J and SM/J genomes using alignments built using the TrEMBL 39.8 protein database. Deleterious amino acid changing mutations were predicted for both strains using a score of < 0.05 and a median conservation cutoff of 3.25. Regulatory annotations are based on the Ensembl regulatory build, which integrates data from ENCODE and several other large scale projects. Transcription factor binding site (TFBS) variant scoring is provided for regulatory regions that have ChIP-seq data to support binding predictions. This is done using the MOODS software [97], which assigns significance scores by matching polymorphisms against motifs in the JASPAR database [98]. The output was filtered for TFBS sites that are classified as ‘Highly Informative’ by the software.

### IBD region identification

To identify regions of shared ancestral background, we clustered segments of observed polymorphisms using a two state hidden Markov model. For each state, we modeled two types of observations: 1) the number of polymorphisms in a 50kbp window; and 2) the observation of a SV in a 50kbp window. The count of polymorphisms is expressed as a Poisson variable, while the occurrence of a SV is a Binomial variable. Parameters for this model were estimated using the EM algorithm implemented in the depmixS4 package in the R programming language [99]. A Wald-Wolfowitz test of randomness was performed using the adehabitatLT package in R [100].

### Data integration

The QTL interrogated in this study are from previously published studies that mapped to the mm9 (NCBI build 37) mouse reference genome. QTL peaks and support intervals were converted to mm10 (GRC m38) using the Batch Coordinate Conversion (liftOver) tool [101]. Sequence data and results generated here were integrated with other published and publically available data as indicated using custom python and R scripts.

### Data access

We have made these data available to the community through multiple data portals: LG/J (SAMN03075510) and SM/J (SAMN03075514) raw reads have been submitted to the NCBI Sequence Read Archive and BAM files have been submitted to the Wellcome Trust mouse genomes portal (http://www.sanger.ac.uk/resources/mouse/genomes). Other results from this study are available as Additional files 1, 2, 3, 4, 5, 6, 7, 8, 9, 10, 11, 12, 13, 14, and 15.

## Additional files

Additional file 1:
**LG/J, SM/J and LG/J x SM/J polymorphic SNPs overview.**
Additional file 2:
**LG/J, SM/J and LG/J x SM/J polymorphic indels overview.**
Additional file 3:
**SNPs in LG/J discovered by integrating output from SamTools pileup and FreeBayes variant discovery tools.**
Additional file 4:
**Indels in LG/J discovered by integrating output from SamTools pileup and FreeBayes variant discovery tools.**
Additional file 5:
**SNPs in SM/J discovered by integrating output from SamTools pileup and FreeBayes variant discovery tools.**
Additional file 6:
**Indels in SM/J discovered by integrating output from SamTools pileup and FreeBayes variant discovery tools.**
Additional file 7:
**Structural variants found in LG/J and SM/J.**
Additional file 8:
**Distances between the LG/J and SM/J strains and other sequenced strains.**
Additional file 9:
**Phylogenetic tree of 20 sequenced mouse strains.**
Additional file 10:
**Amino acid changing mutations and corresponding deleterious predicted scores using SIFT/polyphen/LRT for both LG/J and SM/J strains.**
Additional file 11:
**VEP output of variants in predicted regulatory features for both LG/J and SM/J.**
Additional file 12:
**VEP output of variants in predicted motifs for both LG/J and SM/J.**
Additional file 13:
**Predicted IBD regions in LG/J and SM/J sequenced genomes.**
Additional file 14:
**QTL metrics and sequence integration.**
Additional file 15:
**Distribution of the number of variants predicted to have functional consequences in QTL intervals and in 1000 sets of randomly selected non-QTL regions of similar size.**

## Abbreviations

SNVs: Small nucleotide variants
SVs: Structural variants
IBD: Identical-by-descent
TFBS: Transcription factor binding sites
QTL: Quantitative trait loci
candidate QTG: Quantitative trait gene
QTN: Quantitative trait nucleotide
SNPs: Single nucleotide polymorphisms
Indels: Insertion-deletions
LRT: Likelihood ratio test
FDR: False discovery rate
