# Supplementary material for: Using whole-genome sequences of the LG/J and SM/J inbred mouse strains to prioritize quantitative trait genes and nucleotides
Source: BMC Genomics. 2015 May 28;16(1):415. doi: 10.1186/s12864-015-1592-3 (PMC4445795; doi:10.1186/s12864-015-1592-3)
Supplement: Additional file 1: — LG/J, SM/J and LG/J x SM/J polymorphic SNPs overview. [file 12864_2015_1592_MOESM1_ESM.pdf]

Additional File 1. LG/J, SM/J and LG/J x SM/J Polymorphic SNPs Overview

Chr	total	2.5kb downstream	2.5kb upstream	3prime UTR	5prime UTR	Intron	Regulatory Features	Synonymous	Non-Synonymous	Stop	Splice	Strain*
1	430411	18687	16369	5288	2341	157434	16752	2829	1321	2	0	LG/J
	425372	18768	17414	5568	2251	154505	17621	3103	1458	2	0	SM/J
	439912	16930	18990	4861	1966	151340	15910	2438	1254	2	0	P
2	241268	15301	12729	3751	2107	96344	10082	2081	975	0	1	LG/J
	351593	25530	21520	5766	3846	148851	14934	3642	1929	5	1	SM/J
	324056	25899	21552	5983	3971	150368	13681	3529	1806	5	2	P
3	257379	9487	7716	2445	1290	66540	7698	1293	686	3	1	LG/J
	326406	13015	10908	3161	1742	91204	11400	2179	1076	6	1	SM/J
	291799	10995	9253	2974	1452	90510	9452	1814	926	7	0	P
4	319898	26925	25289	6591	3784	146387	23622	1943	3844	11	1	LG/J
	340286	27243	26054	6713	4024	149744	24518	3905	1916	9	2	SM/J
	303559	23729	21798	5532	3448	139317	17449	2748	1458	6	1	P
5	222406	12179	11414	3011	1919	82164	13374	2045	843	3	0	LG/J
	217450	10483	8961	2651	1498	82436	10582	1739	801	7	0	SM/J
	178882	11146	9647	2910	1646	73929	11098	1708	731	8	0	P
6	254980	12189	11776	3230	1215	94444	7075	1771	1072	3	0	LG/J
	241821	11302	11213	2996	1610	100359	9773	1768	1033	8	0	SM/J
	341520	17152	16807	4190	2066	137252	13377	2209	1313	7	0	P
7	220182	14274	13760	3275	1669	67898	10553	2285	1393	9	0	LG/J
	340882	24159	22923	5125	2897	123357	16221	3897	2194	10	0	SM/J
	349017	27675	26484	5642	3055	127513	16368	3978	2490	18	0	P
8	324513	14733	14011	3691	2237	105646	17088	2577	1243	4	1	LG/J
	321785	15028	13942	3849	2161	104952	16593	2494	1137	4	1	SM/J
	174707	9309	9407	2283	804	78691	11120	1672	723	0	2	P
9	297724	14387	12644	3405	1858	115556	14415	2452	1036	2	0	LG/J
	223192	12052	10680	2976	1471	97299	12377	2160	883	2	0	SM/J
	280513	13520	11959	3343	1630	113360	12638	1974	885	4	0	P
10	217079	8784	7958	2392	1243	74342	9632	1532	653	7	0	LG/J
	193494	10331	8773	2896	1068	71508	11269	1737	702	2	1	SM/J
	270167	12593	11075	3319	1654	90831	14126	2113	845	7	1	P
11	258660	22100	20020	5513	2879	112860	18341	4219	1640	11	1	LG/J
	215773	18146	17015	4621	2392	87800	15667	3816	1210	7	5	SM/J

	225672	22567	20331	5632	2988	99708	17124	4251	1595	14	4	P
	282264	11880	10767	1951	828	70352	10993	1372	538	7	1	LG/J
12	285957	12049	10951	1686	761	66383	10579	1206	523	2	0	SM/J
	279780	13333	12669	1509	860	64411	9605	1013	513	7	1	P
	306024	9916	8555	2548	1152	93365	11506	1494	841	5	0	LG/J
13	224841	9436	8392	2515	1140	79873	10698	1355	701	5	0	SM/J
	266487	9958	8998	2846	1255	76874	11100	1347	624	2	0	P
	177008	8951	8631	1572	961	54225	7075	1301	583	2	0	LG/J
14	301057	10926	10281	1889	1298	74235	9457	1544	781	3	0	SM/J
	191230	6437	6315	1011	667	38234	5069	547	329	1	0	P
	150048	5187	4379	1362	640	43364	6589	718	449	1	0	LG/J
15	148361	5743	4445	1685	668	47242	7252	949	562	0	0	SM/J
	192313	6797	5660	1807	760	61819	8116	1043	446	1	0	P
	127831	4433	3793	1103	696	34994	3743	660	372	0	2	LG/J
16	157397	6950	6510	1769	981	52037	6049	1166	741	2	2	SM/J
	140733	7062	6054	1903	1001	53085	5365	1124	700	2	0	P
	188558	14875	14197	4096	2024	71072	12410	2583	1359	7	0	LG/J
17	166875	11390	10625	2957	1797	64746	10607	2051	955	3	0	SM/J
	191038	14841	14259	3923	2297	73497	12337	2441	1353	10	0	P
	118520	3146	2950	1023	490	41015	4613	512	193	0	0	LG/J
18	145502	4750	4446	1471	646	50568	6325	808	334	0	0	SM/J
	160686	5151	4909	1630	579	58722	6892	787	317	0	0	P
	138869	5806	5201	1842	720	63567	7400	1144	403	1	0	LG/J
19	112000	4460	4137	1496	536	50471	6054	1029	435	2	0	SM/J
	116478	3916	3542	1122	409	50291	5178	817	272	0	0	P
	130101	4502	4410	991	738	20302	1590	241	247	3	0	LG/J
X	52524	2849	2688	716	425	14417	1221	224	178	2	0	SM/J
	129008	4545	4412	989	682	21830	1710	289	217	1	0	P
	4663723	237742	216569	59080	30791	1611871	217714	35052	19691	81	8	LG/J
genome	4792568	254610	231878	62506	33212	1711987	229197	40772	19549	81	13	SM/J
	4847557	263555	227030	63409	33190	1751582	217715	37842	18797	102	11	P

* LG/J = SNPs in LG/J relative to reference genome; SM/J = SNPs in SM/J relative to reference genome; P = SNPs that are polymorphic between the LG/J and SM/J strains.
