# Supplementary material for: Using whole-genome sequences of the LG/J and SM/J inbred mouse strains to prioritize quantitative trait genes and nucleotides
Source: BMC Genomics. 2015 May 28;16(1):415. doi: 10.1186/s12864-015-1592-3 (PMC4445795; doi:10.1186/s12864-015-1592-3)
Supplement: Additional file 2: — LG/J, SM/J and LG/J x SM/J polymorphic indels overview. [file 12864_2015_1592_MOESM2_ESM.pdf]

Additional File 2. LG/J, SM/J and LG/J x SM/J Polymorphic Indels Overview

Chr	total	2.5kb downstream	2.5kb upstream	3prime UTR	5prime UTR	Intron	Regulatory Features	Frameshift	Stop	Splice	Strain*
1	100594	5880	5313	1516	525	47774	1959	16	2	1	LG/J
	97603	5677	5491	1524	530	46237	2032	19	2	0	SM/J
	115599	5121	5046	1327	387	44405	2272	23	0	1	P
2	61777	5729	4749	1135	506	32069	1231	14	1	0	LG/J
	84426	8589	7211	1655	890	46184	1788	18	3	0	SM/J
	86718	7730	6602	1438	698	42149	1836	18	2	0	P
3	61635	3154	2718	755	298	22388	1019	11	4	1	LG/J
	72985	3949	3612	869	428	27716	1423	17	2	5	SM/J
	76029	3304	2979	783	297	25368	1416	14	2	4	P
4	75437	8730	8222	1779	786	44595	2599	32	3	8	LG/J
	78219	8617	8251	1778	833	45213	2574	22	9	0	SM/J
	82338	7347	6804	1347	596	39272	2420	39	6	8	P
5	62543	4624	4684	978	495	31339	1874	5	0	2	LG/J
	57964	3932	3630	900	392	28940	1435	13	0	3	SM/J
	60505	3982	3811	804	397	26602	1815	12	0	1	P
6	59329	3748	3684	877	315	29776	1308	15	0	1	LG/J
	56313	3578	3414	806	387	29777	1294	14	2	1	SM/J
	79445	4213	4173	907	395	34661	1829	23	2	0	P
7	55249	4903	4926	960	388	24838	1355	47	2	0	LG/J
	73576	7086	6807	1240	580	36654	1810	54	3	0	SM/J
	83581	6985	6748	1158	499	34145	2085	57	2	0	P
8	70390	4496	4492	1077	521	31152	1845	14	2	4	LG/J
	69194	4723	4483	1128	499	30588	1822	18	2	7	SM/J
	51456	2857	2900	590	204	22235	1473	7	0	3	P
9	69270	5190	4721	1034	466	36758	1672	18	3	2	LG/J
	57676	4612	4202	939	394	32386	1528	20	2	1	SM/J
	74602	4158	3877	919	344	32499	1778	16	4	1	P
10	55669	3217	3079	780	299	25797	1256	13	1	1	LG/J
	46995	3233	2997	828	282	22810	1257	7	0	0	SM/J
	69279	3607	3434	838	308	26692	1764	15	1	1	P
11	68457	7966	7673	1661	741	39667	2315	43	0	1	LG/J
	56164	6327	6399	1359	586	30906	1968	23	2	0	SM/J
	71359	7623	7360	1498	585	34539	2570	36	2	1	P

12	60501	3173	3028	569	229	22409	1232	7	0	0	LG/J
	59728	3029	2885	537	189	21107	1170	8	9	1	SM/J
	68945	2820	2876	485	191	20467	1336	10	9	1	P
13	65598	3103	2782	727	255	27246	1213	6	0	0	LG/J
	51833	2908	2751	711	266	23494	1232	10	1	0	SM/J
	65268	2846	2630	689	220	21704	1436	6	1	0	P
14	40916	2731	2688	468	238	17474	810	5	0	0	LG/J
	60065	3103	3114	539	306	22052	1003	9	2	0	SM/J
	45675	1848	1948	254	152	13032	754	6	2	0	P
15	39254	2002	1857	485	163	15820	870	15	0	1	LG/J
	38501	2038	1917	527	171	16370	915	10	0	3	SM/J
	52395	2160	2071	509	159	18843	1129	15	0	4	P
16	33445	1600	1344	320	151	12360	538	6	0	0	LG/J
	37846	2262	2053	492	217	16328	806	6	0	0	SM/J
	41128	2184	1898	477	202	16175	831	8	0	0	P
17	45761	4686	4590	1019	449	23535	1430	15	0	1	LG/J
	40884	3921	3808	786	444	21582	1324	12	0	1	SM/J
	50108	4251	4162	838	417	21628	1723	21	0	2	P
18	33362	1291	1239	331	139	14889	608	3	0	0	LG/J
	38159	1648	1612	451	185	17346	855	2	0	0	SM/J
	46100	1635	1650	421	159	17804	987	6	0	0	P
19	34074	2016	1999	575	161	20401	945	3	0	1	LG/J
	27450	1579	1612	475	141	16026	769	3	0	1	SM/J
	30672	1267	1314	345	87	14404	788	4	1	0	P
X	33811	1872	1869	332	195	9253	222	4	0	1	LG/J
	21877	1537	1528	280	146	8181	216	7	0	0	SM/J
	36254	1816	1747	298	165	9698	276	5	1	0	P
genome	1127072	80111	75657	17378	7320	529540	26301	292	18	25	LG/J
	1127458	82348	77777	17824	7866	539897	27221	292	39	23	SM/J
	1287456	77754	74030	15925	6462	516322	30518	341	35	27	P

* LG/J = Indel in LG/J relative to reference genome; SM/J = Indel in SM/J relative to reference genome; P = Indel that are polymorphic between the LG/J and SM/J strains.
