# Supplementary material for: Using whole-genome sequences of the LG/J and SM/J inbred mouse strains to prioritize quantitative trait genes and nucleotides
Source: BMC Genomics. 2015 May 28;16(1):415. doi: 10.1186/s12864-015-1592-3 (PMC4445795; doi:10.1186/s12864-015-1592-3)
Supplement: Additional file 8: — Distances between the LG/J and SM/J strains and other sequenced strains. [file 12864_2015_1592_MOESM8_ESM.pdf]

Additional File 8: Distances Between the LG/J and SM/J Strains and Other Sequenced Mouse Strains

Chr	129P2	129S1	129S5	A/J	AKR/J	BALBc/J	C3HHe/J	C57BL/6NJ	CAST/EiJ	CBA/J	DBA2/J	FVBN/J	LP/J	NODShiLt/J	NZOHlLt/J	PWKPh/J	SPRETEi/J	WSBEi/J	SM/J	LG/J	Strain
1	0.91	0.91	0.91	0.92	0.91	0.90	0.93	0.92	0.67	0.92	0.89	0.91	0.90	0.92	0.90	0.69	0.34	0.88	0.92	1.00	LG/J
	0.91	0.91	0.91	0.92	0.90	0.90	0.91	0.92	0.67	0.91	0.89	0.92	0.91	0.92	0.91	0.69	0.34	0.88	1.00	0.92	SM/J
2	0.92	0.92	0.92	0.94	0.93	0.92	0.92	0.94	0.70	0.93	0.91	0.92	0.91	0.91	0.91	0.66	0.34	0.88	0.93	1.00	LG/J
	0.92	0.92	0.91	0.93	0.92	0.93	0.91	0.92	0.69	0.93	0.91	0.92	0.92	0.91	0.91	0.66	0.34	0.89	1.00	0.93	SM/J
3	0.92	0.92	0.92	0.92	0.96	0.94	0.92	0.94	0.67	0.92	0.94	0.93	0.92	0.94	0.93	0.67	0.34	0.90	0.93	1.00	LG/J
	0.92	0.92	0.92	0.92	0.92	0.93	0.93	0.92	0.68	0.92	0.92	0.91	0.92	0.91	0.91	0.68	0.34	0.89	1.00	0.93	SM/J
4	0.89	0.89	0.89	0.91	0.94	0.92	0.94	0.92	0.66	0.93	0.92	0.92	0.88	0.92	0.91	0.67	0.34	0.88	0.93	1.00	LG/J
	0.88	0.88	0.88	0.90	0.91	0.90	0.92	0.92	0.66	0.92	0.92	0.91	0.87	0.90	0.90	0.67	0.35	0.88	1.00	0.93	SM/J
5	0.93	0.93	0.92	0.96	0.96	0.95	0.94	0.94	0.67	0.95	0.94	0.95	0.93	0.93	0.92	0.65	0.35	0.90	0.96	1.00	LG/J
	0.92	0.92	0.92	0.95	0.95	0.94	0.94	0.95	0.67	0.94	0.93	0.94	0.92	0.92	0.92	0.65	0.35	0.89	1.00	0.96	SM/J
6	0.91	0.91	0.91	0.95	0.94	0.94	0.94	0.94	0.66	0.93	0.95	0.90	0.91	0.90	0.90	0.67	0.35	0.86	0.92	1.00	LG/J
	0.91	0.91	0.91	0.91	0.88	0.91	0.92	0.94	0.65	0.92	0.91	0.92	0.91	0.89	0.87	0.66	0.35	0.87	1.00	0.92	SM/J
7	0.85	0.91	0.91	0.95	0.95	0.94	0.91	0.94	0.65	0.91	0.87	0.93	0.90	0.91	0.90	0.67	0.36	0.89	0.90	1.00	LG/J
	0.81	0.86	0.86	0.89	0.89	0.89	0.87	0.90	0.66	0.87	0.86	0.89	0.86	0.87	0.88	0.69	0.36	0.86	1.00	0.90	SM/J
8	0.92	0.92	0.92	0.92	0.94	0.94	0.92	0.90	0.66	0.95	0.94	0.94	0.92	0.94	0.90	0.66	0.35	0.89	0.95	1.00	LG/J
	0.91	0.91	0.91	0.92	0.93	0.93	0.92	0.90	0.66	0.95	0.94	0.94	0.93	0.93	0.91	0.67	0.35	0.88	1.00	0.95	SM/J
9	0.89	0.89	0.89	0.92	0.90	0.92	0.92	0.90	0.68	0.89	0.90	0.90	0.89	0.92	0.92	0.68	0.35	0.87	0.91	1.00	LG/J
	0.92	0.92	0.92	0.93	0.91	0.93	0.94	0.93	0.68	0.90	0.92	0.92	0.91	0.93	0.90	0.66	0.35	0.90	1.00	0.91	SM/J
10	0.94	0.94	0.94	0.95	0.94	0.95	0.94	0.94	0.65	0.93	0.93	0.92	0.94	0.93	0.93	0.65	0.34	0.88	0.93	1.00	LG/J
	0.93	0.94	0.94	0.94	0.94	0.93	0.95	0.94	0.65	0.95	0.93	0.93	0.94	0.93	0.93	0.66	0.34	0.88	1.00	0.93	SM/J
11	0.91	0.91	0.91	0.90	0.93	0.92	0.90	0.91	0.66	0.91	0.89	0.92	0.92	0.91	0.88	0.67	0.35	0.90	0.93	1.00	LG/J
	0.93	0.93	0.93	0.90	0.91	0.93	0.90	0.93	0.66	0.93	0.91	0.93	0.92	0.92	0.88	0.66	0.35	0.90	1.00	0.93	SM/J
12	0.90	0.90	0.90	0.91	0.92	0.90	0.92	0.91	0.66	0.91	0.90	0.89	0.90	0.91	0.91	0.66	0.36	0.87	0.92	1.00	LG/J
	0.90	0.90	0.90	0.90	0.90	0.91	0.91	0.91	0.66	0.90	0.90	0.90	0.90	0.90	0.91	0.66	0.36	0.88	1.00	0.92	SM/J
13	0.90	0.90	0.90	0.89	0.91	0.91	0.92	0.90	0.66	0.91	0.92	0.91	0.92	0.90	0.89	0.70	0.34	0.87	0.92	1.00	LG/J
	0.92	0.93	0.92	0.90	0.90	0.93	0.94	0.93	0.65	0.95	0.95	0.92	0.91	0.91	0.88	0.68	0.35	0.89	1.00	0.92	SM/J
14	0.87	0.87	0.86	0.94	0.94	0.94	0.91	0.94	0.66	0.92	0.89	0.91	0.81	0.88	0.88	0.78	0.34	0.88	0.94	1.00	LG/J
	0.84	0.84	0.84	0.93	0.94	0.93	0.91	0.90	0.66	0.92	0.89	0.92	0.80	0.91	0.87	0.75	0.34	0.88	1.00	0.94	SM/J
15	0.88	0.88	0.88	0.93	0.94	0.94	0.93	0.94	0.68	0.92	0.93	0.94	0.89	0.92	0.92	0.68	0.34	0.90	0.93	1.00	LG/J
	0.88	0.88	0.87	0.93	0.94	0.93	0.93	0.95	0.68	0.93	0.94	0.94	0.88	0.93	0.93	0.68	0.34	0.89	1.00	0.93	SM/J
16	0.95	0.95	0.94	0.96	0.96	0.97	0.94	0.95	0.67	0.92	0.95	0.96	0.95	0.95	0.95	0.65	0.34	0.90	0.95	1.00	LG/J
	0.92	0.92	0.92	0.93	0.94	0.94	0.92	0.94	0.68	0.92	0.94	0.92	0.93	0.93	0.93	0.66	0.34	0.90	1.00	0.95	SM/J
17	0.91	0.91	0.90	0.93	0.92	0.94	0.91	0.93	0.68	0.91	0.92	0.91	0.92	0.89	0.92	0.66	0.34	0.88	0.93	1.00	LG/J
	0.89	0.89	0.89	0.92	0.92	0.92	0.94	0.93	0.68	0.93	0.92	0.91	0.90	0.89	0.90	0.67	0.34	0.87	1.00	0.93	SM/J
18	0.91	0.91	0.91	0.95	0.94	0.94	0.95	0.95	0.65	0.93	0.95	0.94	0.91	0.94	0.92	0.65	0.35	0.91	0.93	1.00	LG/J
	0.90	0.90	0.89	0.93	0.92	0.92	0.93	0.94	0.65	0.93	0.93	0.93	0.90	0.92	0.91	0.65	0.35	0.90	1.00	0.93	SM/J
19	0.92	0.92	0.92	0.92	0.93	0.91	0.92	0.91	0.67	0.91	0.90	0.91	0.92	0.93	0.93	0.66	0.35	0.89	0.93	1.00	LG/J
	0.93	0.93	0.93	0.92	0.93	0.92	0.92	0.93	0.67	0.91	0.90	0.91	0.93	0.93	0.90	0.65	0.35	0.89	1.00	0.93	SM/J
X	0.95	0.95	0.95	0.98	0.96	0.98	0.98	0.96	0.73	0.98	0.96	0.96	0.95	0.95	0.94	0.70	0.27	0.92	0.96	1.00	LG/J
	0.98	0.98	0.98	0.95	0.99	0.94	0.95	0.98	0.76	0.95	0.99	0.98	0.98	0.98	0.97	0.68	0.27	0.95	1.00	0.96	SM/J
G*	0.91	0.91	0.91	0.93	0.94	0.93	0.93	0.93	0.67	0.92	0.92	0.92	0.91	0.92	0.91	0.67	0.34	0.89	0.93	1.00	LG/J
	0.91	0.91	0.91	0.92	0.92	0.92	0.92	0.93	0.67	0.93	0.92	0.92	0.91	0.92	0.91	0.67	0.34	0.89	1.00	0.93	SM/J

*G = Genome
