# Supplementary figures and images for: Using whole-genome sequences of the LG/J and SM/J inbred mouse strains to prioritize quantitative trait genes and nucleotides
Source: BMC Genomics. 2015 May 28;16(1):415. doi: 10.1186/s12864-015-1592-3 (PMC4445795; doi:10.1186/s12864-015-1592-3)

## Cluster Dendrogram

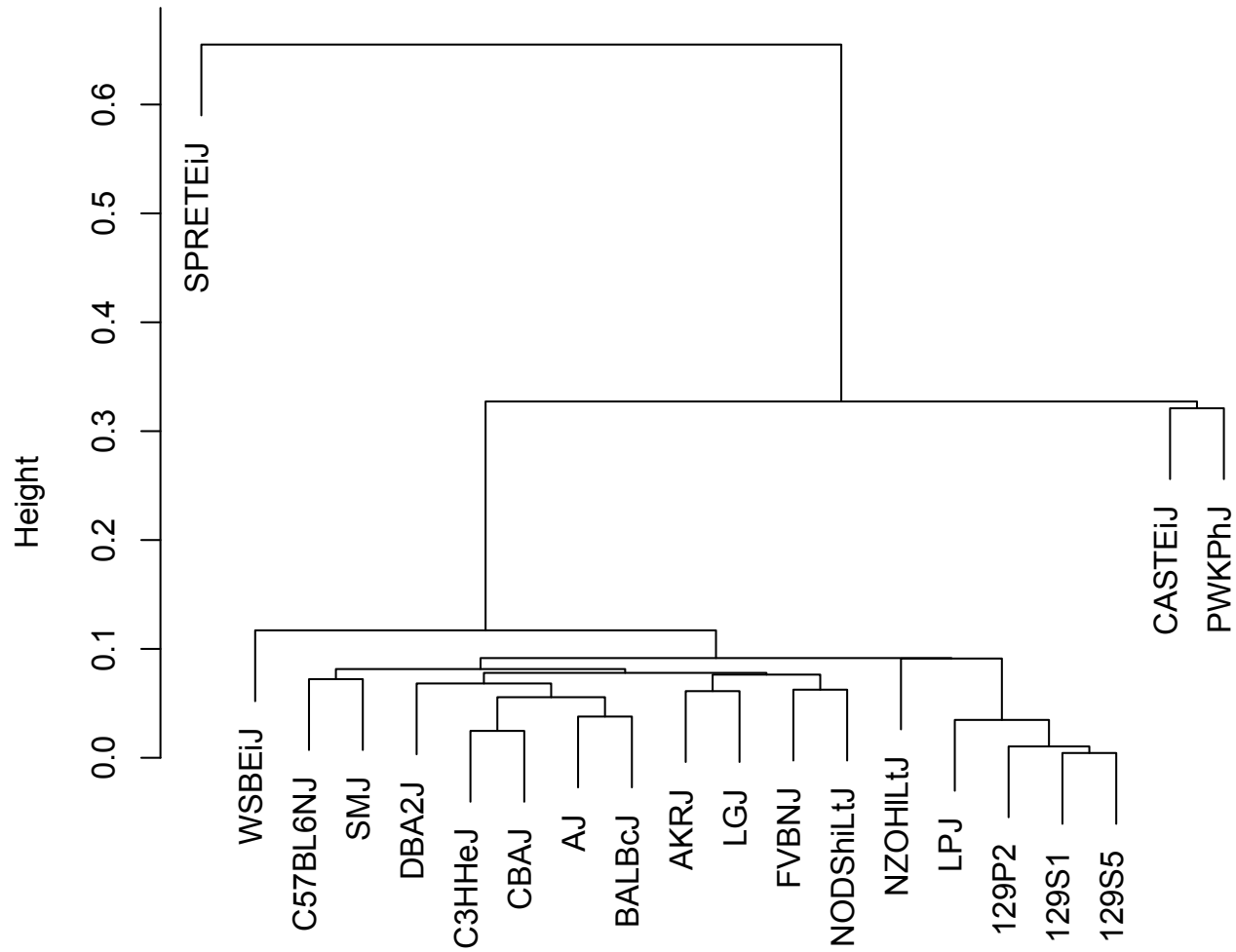

Additional File 9: Phylogenetic Tree of 20 Sequenced Mouse Strains

Supplement: Additional file 9: — Phylogenetic tree of 20 sequenced mouse strains. [file 12864_2015_1592_MOESM9_ESM.pdf]
