# Supplementary material for: Using whole-genome sequences of the LG/J and SM/J inbred mouse strains to prioritize quantitative trait genes and nucleotides
Source: BMC Genomics. 2015 May 28;16(1):415. doi: 10.1186/s12864-015-1592-3 (PMC4445795; doi:10.1186/s12864-015-1592-3)
Supplement: Additional file 15: — Distribution of the number of variants predicted to have functional consequences in QTL intervals and in 1000 sets of randomly selected non-QTL regions of similar size. [file 12864_2015_1592_MOESM15_ESM.pdf]

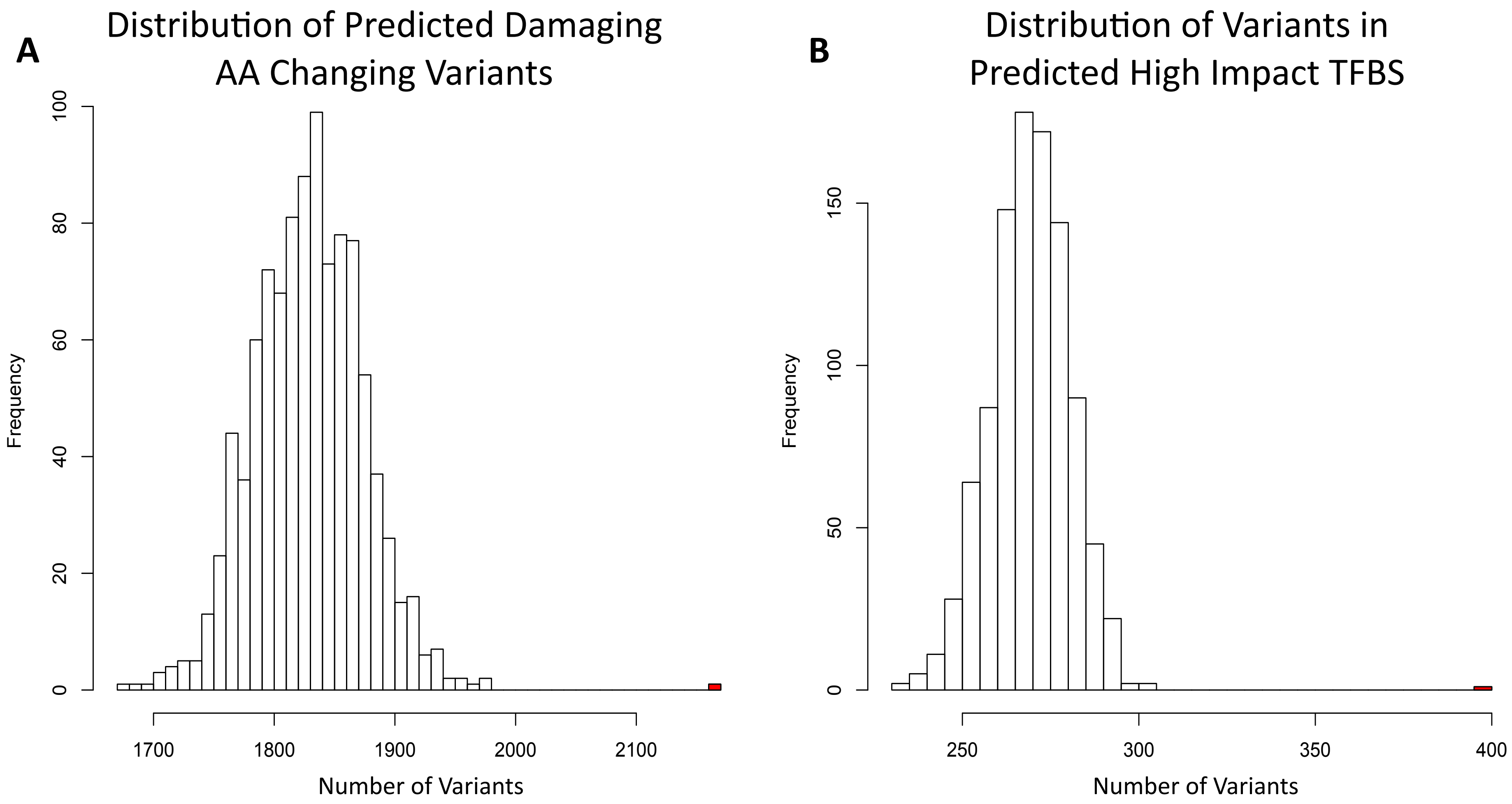

Additional File 15: Distribution of the number of variants predicted to have functional consequences in QTL intervals (indicated by the red bar) and in 1000 sets of randomly selected non-QTL regions of similar size. **A.** The distribution of amino-acid changing mutations predicted to be damaging by at least one algorithm (LRT, PolyPhen, SIFT); $p < 2.2e^{-16}$. **B.** The distributions of variants falling in transcription factor binding site motifs that are predicted to have a high impact (predictions generated by MOODS software); $p < 2.2e^{-16}$.
